# Bone marrow CD34+ molecular chimerism as an early predictor of relapse after allogeneic stem cell transplantation in patients with acute myeloid leukemia

**DOI:** 10.3389/fonc.2023.1133418

**Published:** 2023-03-06

**Authors:** Michele Malagola, Nicola Polverelli, Alessandra Beghin, Federica Bolda, Marta Comini, Mirko Farina, Enrico Morello, Vera Radici, Eugenia Accorsi Buttini, Simona Bernardi, Federica Re, Alessandro Leoni, Davide Bonometti, Duilio Brugnoni, Arnalda Lanfranchi, Domenico Russo

**Affiliations:** ^1^ Blood Diseases and Cell Therapies unit, Bone Marrow Transplant Unit, “ASST-Spedali Civili” Hospital of Brescia, Department of Clinical and Experimental Sciences, University of Brescia, Brescia, Italy; ^2^ Stem Cell Laboratory, Section of Hematology and Blood Coagulation, Clinical Chemistry Laboratory, Diagnostics Department, ASST Spedali Civili of Brescia, Brescia, Italy; ^3^ Centro di Ricerca Emato-oncologico AIL (CREA) , “ASST-Spedali Civili” Hospital of Brescia, Brescia, Italy; ^4^ Department of Hematology, Fondazione IRCCS Ca’ Granda Ospedale Maggiore Policlinico, University of Milan, Milan, Italy; ^5^ Department of Laboratory Diagnostics, ASST Spedali Civili, Brescia, Italy

**Keywords:** *WT1*, allogeneic stem cell transplantation, minimal residual disease (MRD), lineage specific molecular chimerism, pre-emptive therapy

## Abstract

**Background:**

Minimal residual disease (MRD) monitoring is an important tool to optimally address post-transplant management of acute myeloid leukemia (AML) patients.

**Methods:**

We retrospectively analyzed the impact of bone marrow CD34+ molecular chimerism and *WT1* on the outcome of a consecutive series of 168 AML patients submitted to allogeneic stem cell transplantation.

**Results:**

The cumulative incidence of relapse (CIR) was significantly lower in patients with donor chimerism on CD34+ cells ≥ 97.5% and *WT1* < 213 copies/ABL x 10^4 both at 1^st^ month (p=0.008 and p<0.001) and at 3^rd^ month (p<0.001 for both). By combining chimerism and *WT1* at 3^rd^ month, 13 patients with chimerism < 97.5% or *WT1* > 213 showed intermediate prognosis. 12 of these patients fell in this category because of molecular chimerism < 97.5% at a time-point in which *WT1* was < 213.

**Conclusions:**

Our results confirm that lineage-specific molecular chimerism and *WT1* after allo-SCT (1^st^ and 3^rd^ month) are useful MRD markers. When considered together at 3^rd^ month, CD34+ molecular chimerism could represent an earlier predictor of relapse compared to *WT1*. Further studies are necessary to confirm this preliminary observation.

## Introduction

Minimal residual disease (MRD) monitoring is crucial for the management of patients with acute myeloid leukemia (AML) (1–3). Two assays are currently available: multiparametric flow cytometry (MFC) on the leukemia associated immunophenotype (LAIP) and quantitative RT-qPCR on genes known to be mutated or over-expressed in a subgroup of AML (e.g. *FlLT3-ITD*, *NPM1* mutation, *CBF*-fusion transcripts, *WT1* gene,…) (4, 5). Each of these two assays is associated with different specificity, sensitivity and accuracy, and, with the exception of RT-qPCR on *NPM1* mutation, no conclusive data are available on the superiority of one test over the other (4, 6). Nevertheless, several studies confirmed the role of MRD monitoring after induction/consolidation, irrespective of the methods used and the threshold adopted, in order to measure the depth of response during the whole treatment program (3, 4). In particular, it has been suggested that MRD monitoring should be considered as a dynamic event, suggesting that AML risk may be refined during the treatment program (3, 4). Focusing on this issue, we reported how bone marrow (BM) LAIP <0.2% and BM-*WT1* < 121 copies/ABLx10^4 after first consolidation were associated with improved outcome; moreover, after 1^st^ intensification cycle, peripheral blood (PB) *WT1* < 16 copies/ABLx10^4 was significantly correlated with a better prognosis (3). The issue of MRD monitoring is a crucial step in the path to cure of AML patients, especially in low-intermediate ELN risk categories, for which firstline allogeneic stem cell transplantation (Allo-SCT) in case of MRD persistence is a mainstay of good clinical practice (1–4).

Moreover, MRD detection before allo-SCT is very important to guide the intensity of transplant conditioning regimen (7–10). Then, the issue of MRD detection and monitoring after allo-SCT is particularly relevant, since early detection of residual disease may allow a pre-emptive treatment approach, including not only the early immunosuppression withdrawal and donor lymphocytes infusions (DLI), but also the introduction of new drugs such as hypomethylating agents (HMA), venetoclax, and tyrosine-kinase inhibitors (11–13). Although several studies have explored this topic, the methods and timepoints for the detection of patients at high risk of relapse are still a matter of debate (1–4). In particular, besides their limitations in terms of sensitivity and specificity, and the lack of prospective, controlled data, both MFC and RT-qPCR on selected gene targets are applicable in no more than 30-40% of the patients after allo-SCT (4). As a consequence, *WT1* has been suggested as a universal marker of MRD monitoring after allo-SCT, as its expression, although with low specificity, is increased in more than 80% of AML at diagnosis (5, 10, 13).

In this scenario, considering that AML arises from the hematopoietic stem cell, and that more than 90% of AML blasts express CD34 antigen, an option to monitor if allo-SCT has been able to cancel autologous hemopoiesis is the assessment of molecular chimerism on CD34+ cells (14–17). Both short tandem repeat analysis and single nucleotide polymorphism analysis by RT-qPCR have been suggested to be potentially useful tools to measure the degree of donor hematopoiesis. Thus, it may be considered as a surrogate marker of MRD, which can be associated with a high probability of disease recurrence (14–17). Several studies have confirmed that lineage-specific molecular chimerism is a reliable marker of MRD and relapse risk (14–17), but the interplay between CD34+ chimerism and other markers of MRD (e.g., leukemic blasts detection with MFC or *WT1*) possibly associated with MRD persistence is poorly understood and under-studied (18, 19).

With this background, we analyzed a cohort of 168 AML patients consecutively allotransplanted in our Institution between December 2015 and January 2022, for whom at least one between BM-CD34+ chimerism or BM-*WT1* level was available at 1 and 3 months after allo-SCT. The primary endpoint of this retrospective analysis on these two tests was to describe their accuracy in measuring the risk of relapse and their interplay in the definition of patients’ prognosis.

## Patients and methods

From December 2015 to January 2022, a total of 191 AML patients were consecutively submitted to allo-SCT in our Institution. 168 out of these transplants (88%) are included in the present analysis, as they represent a consecutive series for which data on lineage specific molecular chimerism (CD34+) and/or molecular monitoring of *WT1* gene are available at 1^st^ and/or 3^rd^ month after transplant. All patients included in this analysis provided informed consent for data registration in the PROMISE database, in which clinical and biological data are collected. Additional data were extracted from the revision of the clinical charts of each patient, including both the transplant phase and the subsequent follow up. The study was conducted in compliance with current national and European legislation on clinical trials, in accordance with the Declaration of Helsinki and the principles of good clinical practice.

### Lineage-specific chimerism and *WT1* monitoring

According to our guidelines, molecular chimerism assessment on BM-CD34+ cells was planned at months 3, 6, 9, 12, 18, and 24 after allo-SCT. From 2020 we implemented another timepoint of assessment at day +30 after allo-SCT.

CD34+ cells were isolated from bone marrow using CD34 MicroBeads human (Miltenyi Biotec, Bergisch Gladbach, Germany) following manufacturer protocol. Briefly, cells were incubated with 100 µL of CD34 MicroBeads and 100 µL of FcR Blocking Reagent for 30 minutes at 4°C, washed, resuspended in 500 µL buffer and applied onto one-step, semiautomated MACS device, AutoMACS (Miltenyi Biotec, Bergisch Gladbach, Germany). The purity of cellular subsets post-separation was determined by FACS analysis (BD FACSCanto™ II) and BD FACSDiva software (BD Biosciences, San Jose, CA). Genomic DNA obtained after CD34+ selection from BM samples was extracted using mini blood kit (QIAGEN, Valencia, CA), following the manufacturer instructions. Validation of the CD34-enrichment was performed comparing the chimerism percentage of CD34+ and chimerism percentage of MNC between groups by 2-sided Student *t* test (continuous variables with normal distribution). P<.05 was considered significant. Calculations were conducted in Prism 5 (GraphPad, La Jolla, CA). Comparative statistical analysis showed significant difference (P= .0008) and validation of the method. The AmpFLSTR^®^ Identifiler^®^ Plus PCR Amplification Kit (Life Technologies Inc., Foster City, CA) containing 15 polymorphic STR (short tandem repeat) loci and the amelogenin marker was used to evaluate chimerism status in patients post transplant (20). Genomic DNA obtained after CD34+ selection (Automacs System -Miltenyi) from bone marrow samples was extracted using mini blood kit (QIAGEN, Valencia, CA), following the manufacturer instructions. Serial dilutions were created by mixing DNA samples with standardized mixed chimeric samples, in a range between 0% and 100%. The level of sensitivity of this test was 2.5%. The data were analyzed by GeneMapper^®^ID v3.2 software calculating the amount of donor’s DNA.

All the patients with AML were evaluated for *WT1* expression level at diagnosis. Focusing on this series, the time-points of *WT1* evaluation on BM were the same as for chimerism and its assessment was performed by Q-PCR (protocol: Ipsogen *WT1* ProfileQuant) according to the ELN method as previously published (21). The cut-off for positive samples, according to the sensitivity of our platform and available literature, was ≥ 213 *WT1* copies/ABL1x10^4 on BM (21).

### Statistical analysis

Descriptive statistics was employed for summarizing patients characteristics. Categorical variables were presented as numbers and percentages, continuous variables as median and range, respectively. Chi-Squared or Fisher’s Exact test and the Wilcoxon Rank-sum or Kruskal-Wallis tests were used to test differences among subgroups, as appropriate. Median survival with 95% confidence interval (95%CI) was calculated according to reverse Kaplan-Meier method. Overall survival (OS) was measured from the time of transplant to the date of last follow-up or death, cumulative incidence of relapse (CIR), considering non-relapse mortality (NRM) as a competitive event, was carried out according to the Fine-Gray model. Log-rank and Gray tests were employed to verify differences among the different groups. One-month and 3-month landmark analyses were conducted in order to evaluate association between *WT1* (cut-off 213 copies/ABL1x10^4) and donor chimerism (cut-off 97.5%) on subsequent CIR. Sensitivity, specificity and diagnostic accuracy of post-transplant chimerism and *WT1* values in predicting relapse occurrence were also measured. Statistical analysis was performed with EZR (version 1.61), as previously described (22).

## Results

**Table 1** reports the most important clinical and transplant characteristics of these patients. The median age at transplant was 56.5 years (23.8-74.1), and patients were equally distributed between sexes. The disease risk index (DRI) was intermediate/high in two thirds of the cases, and 49.4% of the patients received the transplant in first complete remission (CR) following a myeloablative conditioning in 55.4% of the cases. Peripheral blood stem cells (PBSC) were used in the 76.2% of the cases, and donor was other than a sibling in more than 50% of the transplants (matched unrelated donor in 45.8% and haploidentical in 18.5% of the cases). No significant differences were detected comparing the same characteristics, dividing patients according to the percentage of donor chimerism on CD34+ cells (above or below 97.5%) and *WT1* levels (above or below 213 copies/ABL1x10^4) both at 1^st^ and 3^rd^ month (data not shown).

**Table 1:** Clinical and transplant characteristics of 168 AML patients included in this analysis.

	N (%)
Age, yr, median (range)	56.5 (23.8 – 74.1)
Sex	
Male	91 (54.2)
Female	77 (45.8)
Disease status at SCT	
First CR	83 (49.4)
Other disease status	85 (50.6)
Disease Risk Index	
High - Very	66 (39.3)
Low - Intermediate	102 (60.7)
Follow-up, yr, median (range)	1.5 (0.05 – 14.5)
SC source	
PBSC	128 (76.2)
BM	35 (20.8)
UCB	5 (3.0)
Conditioning intensity	
MAC	93 (55.4)
RIC	75 (44.6)
Donor	
Related	55 (32.7)
MUD	77 (45.8)
Haplo	31 (18.5)
UCB	5 (3.0)
CD34+ donor chimerism (1st month)	*Available on 36 pts*
< 97,5% donor	10 (27.8%)
≥ 97,5% donor	26 (72.2%)
BM *WT1* (1st month)	*Available on 45 pts*
** <** 213 copies/ABLx10^4	11 (24.4%)
≥ 213 copies/ABLx10^4	34 (75.6%)
CD34+ donor chimerism (3rd month)	*Available on 91 pts*
< 97,5% donor	29 (32%)
≥ 97,5% donor	62 (68%)
BM *WT1* (3rd month)	*Available on 120 pts*
** <** 213 copies/ABLx10^4	99 (82.5%)
≥ 213 copies/ABLx10^4	21 (17.5%)
CD34+ donor chimerism/*WT1* (3rd month)	*Available on 75 pts*
≥ 97,5% donor/< 213 copies/ABLx10^4	53 (71%)
< 97,5% donor or ≥ 213 copies/ABLx10^4	13 (17%)
< 97,5% donor and ≥ 213 copies/ABLx10^4	9 (12%)

M, male; F, female; CR, complete remission; DRI, Disease Risk Index; PBSC, peripheral Blood Stem Cells; BM, Bone Marrow; UCB, Umbilical Cord Blood; MAC, Myeloablative Conditioning; RIC, Reduced-Intensity Conditioning; MUD, Matched Unrelated Donor; Haplo, Haploidentical Donor; WT1, Wilm’s Tumor gene.

Overall, the total number of patients for whom *WT1* could be considered for MRD monitoring (at diagnosis > 213) was 125/168 (74%). Molecular chimerism on CD34+ cells and *WT1* at 1^st^ month was available on 36 (21%) and 45 patients (27%), respectively. In 72.2% of the cases (26/36) the percentage of donor CD34+ cells was above 97.5%. Focusing on *WT1*, its level was < 213 copies/ABL1x10^4 in 24.4% of the cases.

Moving to the 3^rd^ month, molecular chimerism on CD34+ cells and *WT1* were available on 99 (53%) and 125 patients (67%), respectively. Donor chimerism ≥ 97.5% was detected in 63 patients (65.6%) and *WT1* levels < 213 copies/ABL1x10^4 in 103 cases (82.4%).

Additional molecular markers of disease persistence during follow up were *FLT3-ITD* (2 cases at 1^st^ month and 4 cases at 3^rd^ month) and *NPM1A* (2 cases at 1^st^ month and 2 cases at 3^rd^ month). All the patients with positive *FlLT3-ITD* MRD had mixed chimerism on CD34+ and *WT1* level above 213 copies/ABL1x10^4, experienced hematological relapse and did not survive. The 2 patients with *NPM1A* positive residual disease showed complete donor chimerism and *WT1* level < 213 copies/ABL1x10^4 and are alive in continuous complete remission at last follow up.

### Cumulative incidence of relapse and overall survival

**Figure 1:**
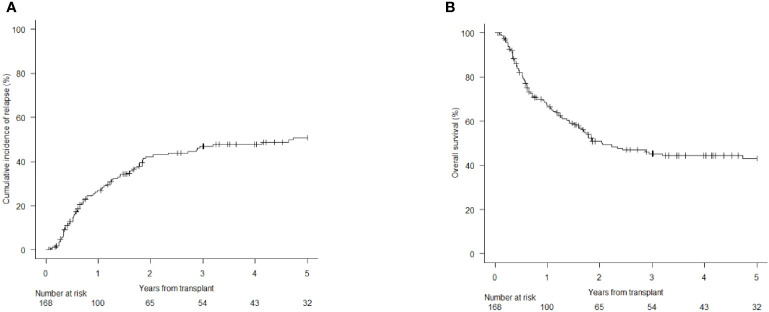
Cumulative Incidence of Relapse (CIR) and Overall Survival (OS) of the 168 AML patients included in this analysis. [CIR at 1, 3 and 5 years; 26.9% (95% CI 20.3-34.0), 46.8% (95% CI 38.6-54.4) and 50.8% (95% CI 42.2-58.9) **(A)**; OS at 1, 3 and 5 years: 67.3% (95% CI 59.4-74), 50.9% (95% CI 42.6-58.6) and 43.2% (95% CI 34.8-51.3) **(B)**].

After a median follow up of 4.5 years (range 3,5-5,0), the 1, 3, and 5 years cumulative incidence of relapse (CIR) was 26.9% (95% CI 20.3-34.0), 46.8% (95% CI 38.6-54.4) and 50.8% (95% CI 42.2-58.9), respectively (**Figure 1A**). This translated into an overall survival (OS) at 1,3 and 5 years of 67.3% (95% CI 59.4-74), 50.9% (95% CI 42.6-58.6), and 43.2% (95% CI 34.8-51.3), respectively (**Figure 1B**).

**Figure 2:**
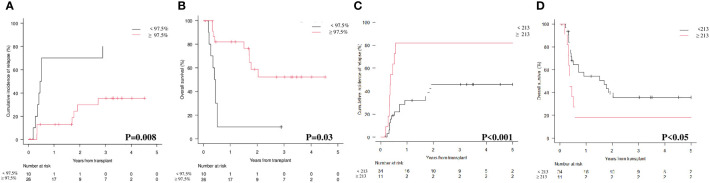
Cumulative Incidence of Relapse (CIR) and Overall Survival (OS) according to molecular chimerism on CD34+ cells and *WT1* levels at 1st month. **(A)** CIR at 1 year CD34+≥97.5% vs <97.5% donor: 13% (95% CI 3.3-29.7) vs 70% (95% CI 32.9.89.2). **(B)** OS at 1 year CD34+≥97.5% vs <97.5% donor: 81.8% (95% CI 58.5-92.8) vs 9.5% (95% CI 5-35.8). **(C)** CIR at 1 year *WT1* < 213 copies/ABL1×10^4≥213 copies/ABL1×10^4: 31.8% (95%CI 16.7-48.2) vs 81.8% (95%CI 44.7-95.1). **(D)** OS at 1 year *WT1* < 213 copies/ABL1×10^4≥213 copies/ABL1×10^4: 54.3% (95%CI 35.3-69.9) vs 18.2% (95%CI 2.8-44.2).

At 1^st^ month, both donor chimerism on CD34+ cells ≥ 97.5% and *WT1* levels below 213 copies/ABL1x10^4 significantly correlated with CIR (chimerism: 13% *vs* 70% at 1 year; p=0.008 – **Figure 2A**; *WT1*: 31.8% *vs* 81.8%; p=0.03 – **Figure 2B**) and OS (chimerism: 81.8% *vs* 9.5% at 1 year; p<0.001 – **Figure 2C**; *WT1*: 54.3% *vs* 18.2%; p<0.05 – **Figure 2D**).

**Figure 3:**
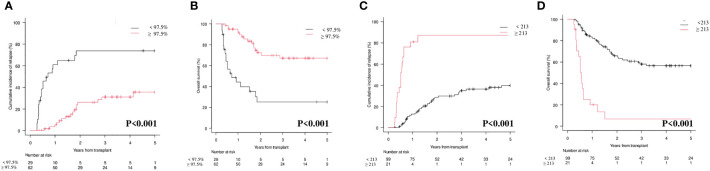
Cumulative Incidence of Relapse (CIR) and Overall Survival (OS) according to molecular chimerism on CD34+ cells and *WT1* levels at 3rd month. **(A)** CIR at 1 year CD34+≥97.5% vs <97.5% donor: 5.3% (95% CI 1.4-13.4) vs 61% (95% CI 40.3-76.4). **(B)** OS at 1 year CD34+≥97.5% vs <97.5% donor: 93.1% (95% CI 82.6-97.3) vs 44.2% (95% CI 25-61.9). **(C)** CIR at 1 year *WT1* < 213 copies/ABL1×10^4≥213 copies/ABL1×10^4: 12.6% (95%CI 6.9-20.2) vs 80.9% (95%CI 56.9-92-4). **(D)** OS at 1 year *WT1* < 213 copies/ABL1×10^4≥213 copies/ABL1×10^4: 83.3% (95%CI 74.2-89.4) vs 20.1% (95%CI 6.2-39.5).

As reported in **Figures 3A, D**, the results at 3^rd^ month confirmed the predictive value of the two markers on CIR and OS. In particular, the 1 and 2 years CIR for patients with donor chimerism on CD34+ cells ≥ 97.5% *vs* those with donor chimerism < 97.5% was 5.3% and 26% *vs* 61% and 74%, respectively (**Figure 3A**; p<0.001). This translated into a 1 and 2 years OS of 93% and 72.4% *vs* 44.2 and 25.4%, respectively (**Figure 3B**; p<0.001). Moving to *WT1* at 3rd month and comparing patients with a level below or above 213 copies/ABL1x10^4, we observed that the CIR at 1 and 2 years was 12.6% and 28.6% *vs* 80.9% and 97.3%, respectively (**Figure 3C**; p<0.001). As expected, 1 and 2 years OS was 83.3% and 65.8% *vs* 20.3% and 6.7% (**Figure 3D**; p<0.001).

The sensitivity, specificity, and accuracy of molecular chimerism on CD34+ cells at 3^rd^ month was 53.3% (95% CI 34.3-71.7), 83.7% (95% CI 70.3-92.7), and 72.2% (85% CI 60.9-81.7). For *WT1* at 3^rd^ month we observed a sensitivity of 33.3% (95% CI 17.3-52.8), a specificity of 98% (95% CI 89.1-99.9) and an accuracy of 73.4% (95% CI 62.3-82.7). We then looked at the sensitivity, specificity and accuracy of the combination of donor chimerism and *WT1* levels at 3^rd^ month and we found that they were 53.3% (95% CI 34.3-71.7), 81.6% (95% CI 68.0-91.2) and 70.9% (95% CI 59.6-80.6).

Interestingly, by combining CD34+ donor chimerism </≥ 97.5% and *WT1* </≥ 213 copies/ABL1x10^4, three categories could be identified with significantly different prognosis both on CIR (p<0.001; **Figure 3A**) and on OS (p<0.001; **Figure 3B**): (i) donor chimerism ≥ 97.5% and *WT1* < 213 (53 patients) [CIR at 1 year 4.1% (95% CI 0.8-12.4) and OS at 1 year 94.2% (95% CI 83.0-98.1)]; (ii) donor chimerism < 97.5 or *WT1* ≥ 213 (13 patients) [CIR at 1 year 30.7% (95% CI 9.5-55.4) and OS at 1 year 76.9% (95% CI 44.2-91.9)]; (iii) donor chimerism < 97.5% and *WT1* ≥ 213 (9 patients) [CIR at 1 year 100% (95% CI NA) and OS at 1 year 0% (95% CI NA)]. Moreover, 12/13 patients included in the “intermediate” group (donor chimerism < 97.5% or *WT1* ≥ 213) fell in this category because of donor chimerism < 97.5% and only 1 patient because of *WT1* ≥ 213 copies/ABL1x10^4.

### Pre-emptive treatment following the detection of CD34+ donor chimerism < 97.5% and/or *WT1* ≥ 200 copies/ABL1x10^4

Overall, 43 and 66 patients had at least one detection of donor CD34+ chimerism < 97.5% and/or *WT1* levels ≥ 213 copies/ABL1x10^4 at 1^st^ and/or 3^rd^ month. Whenever clinically possible (no graft versus host disease and no active infections) these patients were managed with early tapering of immunosuppression. If clinical and hematological conditions were permissive, additional pre-emptive therapy was administered (11 patients). Results in the different subgroups are reported in **Supplementary Table 1**.

## Discussion

Minimal Residual Disease (MRD) monitoring is crucial in the management of AML patients, and is a dynamic process during all the treatment plan, including the post-transplant phase (1–17, 23, 24).

**Figure 4:**
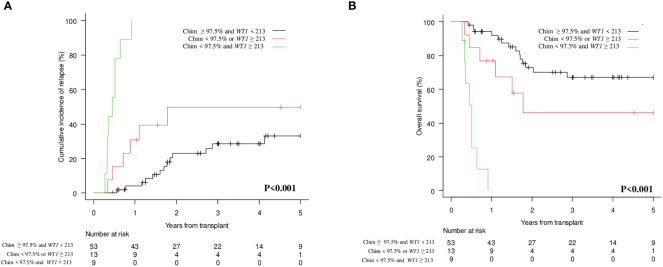
Cumulative Incidence of Relapse (CIR) and Overall Survival (OS) according to combination of molecular chimerism on CD34+ cells and *WT1* levels at 3rd month. **(A)** CIR at 1 year CD34+≥97.5% and *WT1* < 213 copies/ABL1×10^4 vs CD34+ <97.5% *WT1* ≥ 213 copies/ABL1×10^4 vs CD34+ <97.5% or *WT1* ≥ 213 copies/ABL1×10^4: 4.1% (95% CI 0.8-12.4) vs 30.7% (95% CI 9.5-55.4) vs 100% (95% CI NA). **(B)** OS at 1 year CD34+≥97.5% and *WT1* < 213 copies/ABL1×10^4 vs CD34+ <97.5% or *WT1* ≥ 213 copies/ABL1×10^4 vs CD34+ <97.5% and *WT1* ≥ 213 copies/ABL1×10^4: 94.2% (95% CI 83.0-98.1) vs 76.9% (95% CI 44.91.9)] vs 0% (95% CI NA).

Our study clearly shows that both lineage-specific molecular chimerism and *WT1* levels are useful markers for MRD detection and monitoring after allo-SCT in AML, either alone or in combination. At 1^st^ month after allo-SCT, lineage-specific molecular chimerism (**Figure 2A**; p=0.008) and *WT1* levels (**Figure 2C**; p<0.001) were significantly correlated with the CIR. This was also confirmed at 3^rd^ month (**Figure 3A**; p<0.001 and **Figure 3B**; p<0.001). Interestingly, by combining molecular chimerism and *WT1* at 3^rd^ month, we identified three categories of patients with different prognosis: (i) donor chimerism ≥ 97.5% and *WT1* < 213 (53 patients); (ii) donor chimerism < 97.5 or *WT1* ≥ 213 (13 patients); (iii) donor chimerism < 97.5% and *WT1* ≥ 213 (9 patients). The lowest CIR and the longest OS were observed in patients with donor CD34+ ≥ 97.5% and *WT1* < 213 copies/ABL1x10^4 (**Figure 4A**; p<0.001 and **Figure 4B**; p< 0.001). This strongly reinforces the significance of these two tests for MRD monitoring after allo-SCT. Notably, focusing on the intermediate category (donor CD34+ chimerism < 97.5% or *WT1* ≥ 213 copies/ABL1x10^4), we observed that nearly all of these patients (12/13) were included in this group because of mixed donor chimerism, at a timepoint in which *WT1* levels were still within the normal range. This suggests that molecular chimerism may detect persistence of MRD earlier than *WT1*. In other words, once *WT1* gets positive, disease relapse is highly likely to occur in a very short time-frame. Even if numbers are small to be conclusive, we think that these results reinforces the usefulness of both methods for MRD monitoring after allo-SCT and suggests that lineage-specific molecular chimerism may an earlier predictor of relapse than *WT1*. On the other hands, a recently published paper (18) suggests that day +100 MRD positivity is a stronger predictor of relapse after allo-SCT compared to mixed chimerism. Notably, the series published by Klyuchinov and Colleagues includes intermediate-risk AML only, and MRD monitoring was performed with MFC and RT-qPCR on *NPM1A*, of which at least *NPM1A* is extremely disease-specific as a marker of MRD. These two aspects may be responsible for the different results.

We then looked at the use of pre-emptive therapy guided by one or both of the MRD markers (chimerism and/or *WT1*). Pre-emptive treatment was administered in a minority of patients (n=11). As a consequence results are anecdotal and no conclusions can be drawn. Interestingly, the higher response rate (in terms of conversion to full donor chimerism or increase in the percentage of donor CD34+ cells) was observed in patients with mixed chimerism at 3^rd^ months (n=29). In this group, 7 patients (24%) received a pre-emptive approach with either HMA alone or in combination with venetoclax/DLI or DLI alone, 4/7 (57%) patients achieved a response and at the last follow up 9/29 (31%) patients were alive.

The relatively small number of patients included in our analysis may hamper drawing final conclusions. Nevertheless, our study confirms the prognostic value of lineage-specific chimerism at very early timepoints (1^st^ and 3^rd^ month) and suggests that patients at high risk of relapse may show mixed chimerism before positivity of *WT1* as a marker of MRD. The aim of this study was not to compare lineage-specific molecular chimerism and *WT1*, but our results indirectly suggest that chimerism on CD34+ cells could be an earlier predictor of relapse. The small number of cases with available data at day +30 depends on the fact that early assessment of chimerism and MRD monitoring were implemented only from 2020 in our Institution and suggests caution both in results interpretation and conclusion drawing.

Overall, our data are in line with other published papers, highlighting the role of both lineage specific molecular chimerism and *WT1* as markers of MRD after allo-SCT (14–19, 24–28). The issue of the superiority of molecular chimerism on CD34+ cells over other methods for leukemia relapse prediction is still unsolved. Some data suggest that *WT1* could be more sensitive than lineage specific molecular chimerism (29) or that the two methods are concordant (30), also when analyzed in specific cellular sub-types, such as CD3 negative mononuclear cells (31). On the other hand, in the study by Rossi and Colleagues a higher concordance between positive results from MFC and *WT1* was detected among patients with mixed rather than complete chimerism (32). Several issues are still open, such as the role of the source used for the detection of both chimerism and MRD. If it is true that PB may be used for MRD monitoring in AML and has some advantages over bone marrow (13), there are no conclusive data regarding this issue when we consider lineage-specific molecular chimerism and different sources are used in the published papers, according to each Center’s guideline (15, 26–32). Interestingly, as suggested by Gambacorta and Colleagues, PB may allow a tighter follow up of the patients and may allow higher specificity in case of positive samples. The Authors give an intriguing explanation for this, speculating that BM detects a significant “background noise” possibly related to the aspiration of host stromal cells (15). Moreover, new technologies such as digital PCR (dPCR) or next generation sequencing (NGS) may be a useful tool to increase both the specificity and sensitivity of lineage-specific molecular chimerism. Further prospective studies are thus warranted in order to clarify if lineage-specific molecular chimerism is superior to *WT1* to identify imminent relapse, which time-points are more reliable for an optimal prediction of disease recurrence, if PB should be preferred to BM and if new technologies may increase the power of molecular chimerism for relapse prevention.

## Data availability statement

The raw data supporting the conclusions of this article will be made available by the authors, without undue reservation.

## Ethics statement

Ethical review and approval was not required for the study on human participants in accordance with the local legislation and institutional requirements. The patients/participants provided their written informed consent to participate in this study.

## Author contributions

MM, NP, ArLa, DR designed the study. EM, VR, EB, SB, FR, DaBo and AlLe collected the data. ArLa, AB, FB, MC performed chimerism analysis. DuBr performed molecular analysis on WT1. MM, NP, AlLe, ArLa, SB, FR analyzed the data. MM, NP, ArLa and DR wrote the Manuscript. All authors contributed to the article and approved the submitted version.
