# Supplementary material for: Bone marrow CD34+ molecular chimerism as an early predictor of relapse after allogeneic stem cell transplantation in patients with acute myeloid leukemia
Source: Front Oncol. 2023 Mar 6;13:1133418. doi: 10.3389/fonc.2023.1133418 (PMC10025489; doi:10.3389/fonc.2023.1133418)
Supplement: Supplementary file 1 [file Table_1.doc]

**Supplementary Table 1. Patients’ management according to chimerism and/or *WT1* levels during follow up**

| **MRD marker and timepopint** | **N° pts treated** | **Treatment received** | **Outcome at last follow up** |
| --- | --- | --- | --- |
| Molecular chimerism < 97.5% at 1st month (n=10) | 2 | HMA/Ven (1)  Sorafenib/DLI (1) | 2/2 (100%) pts died for disease relapse |
| Molecular chimerism < 97.5% ad 3rd month (n=29) | 7 | HMA/Ven (1)  HMA/DLI (1)  HMA/Ven/DLI (2)  DLI (3) | 4/7 pts achieved complete donor chimersim  9/29 (31%) pts alive and in continuous CR |
| *WT1* ≥ 213 copies/ABL x 10^4 at 3rd month (n=21) | 2 | HMA/Ven (1)  HMA (1) | 2/2 (100%) pts died for disease relapse |

**List of abbreviations:** HMA=Hypo-Methylating Agens; Ven=Venetoclax; DLI=Donor Lymphocyte Infusion
